# Development of lateral flow assays for the rapid diagnosis of glanders in equids

**DOI:** 10.1128/spectrum.00154-26

**Published:** 2026-07-10

**Authors:** Ryo Shimbo, Kazuki Mizuta, Yoshiki Ichikawa, Enkhtuul Batchuluun, Ochirbat Khurtsbaatar, Ai Koshikawa, Yuta Kinoshita, Hidekazu Niwa, Tomohiro Okagawa, Keisuke Aoshima, Vanaabaatar Batbaatar, Kazuhiko Ohashi, Takashi Kimura

**Affiliations:** 1 Laboratory of Comparative Pathology, Faculty of Veterinary Medicine, Hokkaido University, Sapporo, Japan; 2 Department of Small Animal Clinical Sciences, Western College of Veterinary Medicine, University of Saskatchewan, Saskatoon, Canada; 3 Department of Veterinary Forensics, Faculty of Veterinary Medicine, Hokkaido University, Sapporo, Japan; 4 Laboratory of Infectious Disease and Immunology, Institute of Veterinary Medicine 832673, Ulaanbaatar, Mongolia; 5 Equine Research Institute, Japan Racing Association, Tokyo, Japan; 6 Laboratory of Infectious Disease, Faculty of Veterinary Medicine, Hokkaido University, Sapporo, Japan; Ross University School of Veterinary Medicine, Basseterre, Saint Kitts and Nevis

**Keywords:** *Burkholderia mallei*, lateral flow assay, glanders

## Abstract

Glanders is a contagious zoonotic disease caused by *Burkholderia mallei* that primarily affects horses, donkeys, and mules. It remains endemic in parts of Asia, Africa, the Middle East, and South America, where increasing case reports highlight the need for accessible diagnostic tools. Conventional diagnostic methods are limited by high costs and specialized laboratory requirements, whereas lateral flow assays (LFAs) offer practical, user-friendly, and rapid alternatives. In this study, we developed three LFAs targeting antibodies against the *B. mallei* antigens Hcp1, GroEL, and whole-cell lysate (WCL), previously identified as promising markers in indirect enzyme-linked immunosorbent assays. Recombinant streptococcal protein G served as the colloidal gold conjugate. Diagnostic performance was evaluated using glanders-positive (*n* = 31) and glanders-negative (*n* = 51) equine sera collected in Mongolia. Hcp1-LFA demonstrated 100% sensitivity and 100% specificity, while WCL-LFA achieved 100% sensitivity and 98.0% specificity. GroEL-LFA showed 100% sensitivity but lower specificity (90.2%). Hcp1- and WCL-LFA also exhibited high sensitivity in asymptomatic horses. No cross-reactivity was observed with sera from horses with other infections (*n* = 60) collected in Japan. All LFAs remained functional for at least 16 weeks at 37°C. These results indicate that Hcp1- and WCL-based LFAs provide accurate, rapid, and equipment-free detection of glanders. Their simplicity, stability, and affordability make them highly suitable for field diagnosis, especially in low-resource endemic settings. Nonetheless, further validation using larger and more diverse sample sets is required to confirm their broader applicability.

**IMPORTANCE:**

Glanders is a contagious disease affecting horses, donkeys, and mules that can have serious economic and animal health consequences. Current diagnostic tests are expensive, time-consuming, and require specialized equipment. The assays developed in this study are simple, rapid, and cost-effective, allowing veterinarians to quickly identify infected animals in the field. Early detection helps prevent the spread of the disease, safeguarding livestock, supporting animal health, and reducing economic losses in affected regions.

## INTRODUCTION

Glanders is an infectious disease caused by the gram-negative bacterium *Burkholderia mallei*. It primarily affects equids (horses, donkeys, and mules), but it can also infect camels, cats, dogs, wolves, bears, and humans (1). In horses, the disease typically follows a chronic and subclinical course, progressing over months to years after the acute phase and ultimately resulting in death (1). Nasal discharge and cutaneous exudates from horses with glanders are infectious, and transmission occurs through close contact, inhalation, or ingestion of contaminated materials (1).

Glanders has been eradicated in many countries, including the United States, Australia, Canada, and Western Europe, through testing, culling, and quarantine (1–4). However, the disease remains endemic in regions of Asia, Africa, the Middle East, and South America. In recent years, reported cases have increased in countries such as Mongolia (5), India (6–8), Iran (9), and Brazil (10), suggesting a re-emergence consistent with reports from the World Organisation for Animal Health.

In endemic regions, surveillance through the investigation of suspected cases and randomized or targeted sampling is essential for disease control (11). However, current diagnostic methods, such as the complement fixation test (CFT) and polymerase chain reaction (PCR), are hampered by high costs and specialized laboratory facilities. As a result, their use in resource-limited regions is constrained by insufficient funding and limited access to testing laboratories (12). Addressing these challenges requires the development of low-cost, user-friendly, on-site diagnostic tools.

Lateral flow assays (LFAs) offer visually interpretable results within approximately 10 min using only a serum sample. They also provide high stability, allowing 1–2 years of storage at room temperature, require no specialized training, and can be produced at low cost. These characteristics make LFAs highly suitable for deployment in resource-limited regions. Since the bacterium is rarely detectable in blood samples from naturally infected cases (13, 14), detecting the significant rise in antigen-specific antibodies (15) is essential for achieving a reliable diagnosis. In this study, we developed LFAs using three *B. mallei* antigens, Hcp1, GroEL, and whole-cell lysate (WCL), previously identified by our research team as highly immunogenic (16).

## MATERIALS AND METHODS

### Serum samples

Serum samples were classified into four groups (Groups A–D). Samples in Groups A, B, and C were collected between 2018 and 2024 from horses owned by nomadic herders across multiple provinces in Mongolia, including Dornod, Sukhbaatar, Khentii, Tuv, Selenge, Ulaanbaatar, Dundgovi, Umnogobi, Uvurkhangai, Arkhangai, Zavkhan, and Khovd. Group D samples were obtained from Thoroughbred racehorses in Japan, a glanders-free country.

Group A consisted of 24 positive and 36 negative serum samples and was used to optimize the antigen concentrations for the LFA strips. The positive samples were obtained from horses that tested positive by the complement fixation test, and the negative samples were confirmed negative by CFT.

Group B comprised 31 positive and 51 negative serum samples and was used to validate the diagnostic sensitivity and specificity of the LFAs. Glanders-positive horses met all of the following criteria: positive results in the CFT, Mallein test, and ID Screen Glanders Enzyme-linked immunosorbent assay (ELISA) (ID Screen Glanders Double Antigen Multi-species; Innovative Diagnostics, Grabels, France), as well as the presence of characteristic clinical signs (e.g., nasal discharge and skin ulcers). Glanders-negative horses were negative in the CFT and ID Screen ELISA and exhibited no clinical signs.

Group C included 31 positive serum samples from asymptomatic horses that tested positive by both CFT and the ID Screen ELISA. These samples were used to evaluate the LFA performance under conditions simulating field surveillance of subclinical or latent infections.

Group D included 60 sera collected in Japan from Thoroughbred racehorses with conditions other than glanders: fever (*n* = 15), shipping fever (*n* = 15), phlegmon (*n* = 15), and experimental infection with *Streptococcus equi* subsp. *zooepidemicus* (*n* = 3). For experimentally infected horses, sera were collected at 2, 4, 6, 14, and 28 days post-infection.

### Complement fixation test

The CFT was performed as previously described (5). Serum samples from Groups A–C were diluted 1:5 in 0.9% NaCl and heat-inactivated at 56°C for 30 min. The diluted serum, 1:40 diluted guinea pig complement, and CFT antigen (Biocombinat, Ulaanbaatar, Mongolia) were mixed in quadruplicate in 96-well round-bottom plates and incubated at 4°C for 16–18 h. Sensitized sheep red blood cells (2% suspension; 100 µL/well) were added, followed by incubation at 37°C for 45 min and centrifugation at 600 × *g* for 5 min. Hemolysis was assessed visually. Samples showing complete hemolysis in all replicate wells were recorded as negative, whereas those showing no hemolysis in any well were recorded as positive.

### Mallein test

The Mallein test was performed as described previously (5). Horses selected for testing exhibited typical clinical signs and had a positive CFT result. Each horse received an intradermal injection of 0.2 mL (1.0 mg/mL) concentrated Mallein-purified protein derivative (Biocombinat) at the midpoint of the vertical side of the neck. Injection sites were examined at 24, 48, and 72 h post-injection. A test was considered positive when a firm, painful swelling ≥6 mm in diameter was observed at 24 and 48 h.

### ID Screen ELISA

Groups B and C samples were tested using the ID Screen ELISA kit following the manufacturer’s instructions. The cutoff was defined as 70% of the absorbance of the positive control.

### Preparation of recombinant Hcp1, GroEL, and streptococcal protein G

Recombinant *B. mallei* antigens were prepared as previously described (16). Genes encoding Hcp1 (BMAA0742) and GroEL (BMA2001) from *B. mallei* ATCC 23344 were amplified by PCR, digested with the appropriate restriction enzymes, and ligated into linearized pET28a(+) vectors (Merck, Darmstadt, Germany).

Streptococcal protein G (SPG) was selected due to its high affinity for equine IgG (17, 18). The IgG-binding domains (amino acids 190–384; GenBank ID: CAA27638.1) were codon-optimized for *Escherichia coli*, synthesized, and ligated into the pEX-A2J2 vector (Eurofins Genomics, Tokyo, Japan). The insert was excised and ligated into pET28a(+). Expression vectors were transformed into *E. coli* BL21 (DE3) cells. Protein expression was induced with 0.2 mM IPTG at 16°C for 20 h. Cells were harvested by centrifugation (9,000 × *g* for 10 min) and lysed using BugBuster Master Mix (Merck). Soluble fractions were collected following centrifugation and purified using Ni-NTA agarose (FUJIFILM Wako, Osaka, Japan) in Poly-prep columns (Bio-Rad, Hercules, CA, USA). Purity was assessed by sodium dodecyl sulfate-polyacrylamide gel electrophoresis with Coomassie staining (Fig. S1a). Buffers were exchanged to phosphate-buffered saline (PBS, pH 7.4), and proteins were concentrated using centrifugal concentrators. Protein concentrations were determined using the BCA Protein Assay Kit (Takara Bio).

### Preparation of whole-cell lysate

WCL was prepared as previously described (16). All culture and lysis steps were conducted in BSL-3 facilities at Hokkaido University (Japan) and the Institute of Veterinary Medicine (Mongolia). Lysates were released only after confirming the absence of viable bacteria.

*B. mallei* ATCC 23344 was cultured on brain heart infusion agar with 4% glycerol at 37°C for 48 h. Colonies were resuspended in PBS, aliquoted (500 µL), centrifuged (13,000 × *g* for 5 min), and stored at –80°C. For lysis, pellets were resuspended in 4 mL of lysis buffer (50 mM Tris-HCl [pH 8.0], 500 mM NaCl, 1% Triton X-100, 0.5% sodium deoxycholate, 0.1% CHAPS, and 800 µg lysozyme) and incubated on ice with shaking for 30 min. Magnesium sulfate (final 50 mM) and DNase I (8 µL) were added, followed by further incubation on ice. Lysates were centrifuged (9,400 × *g* for 10 min), and supernatants were used as antigen.

### Preparation of lateral flow assay strips

**Fig 1:**
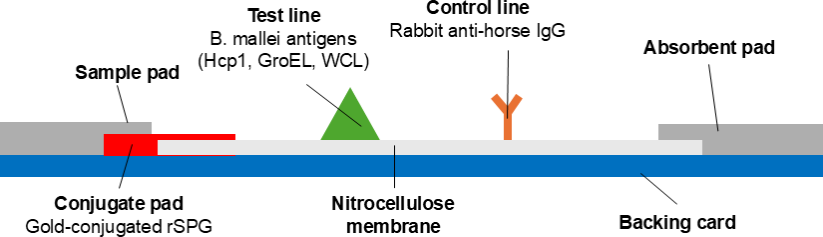
Schematic illustration of the developed LFA strips. The device is designed for the detection of anti-Hcp1, anti-GroEL, or anti-WCL IgG. Hcp1 (2 mg/mL), GroEL (0.1 mg/mL), or WCL (0.2 mg/mL) were immobilized on the test line, and rabbit anti-horse pAb (0.5 mg/mL) was immobilized on the control line. The gold-conjugated recombinant streptococcal protein G (rSPG) was applied to the conjugate pad.

A schematic of the LFAs is shown in Fig. 1. Antigen concentrations were optimized using Group A serum samples to ensure the best diagnostic performance. Antigens (Hcp1, GroEL, and WCL) were diluted in PBS to 2, 0.1, and 0.2 mg/mL, respectively. Polyclonal rabbit anti-horse IgG (Cosmo Bio, Tokyo, Japan) was diluted to 0.5 mg/mL in 50 mM carbonate-bicarbonate buffer (pH 9.6).

Nitrocellulose membranes (HF180; Sigma-Aldrich, Burlington, MA, USA) were striped with antigen (test line) and rabbit anti-horse IgG (control line) using an air-jet dispenser (CyberJet2; Musashi Engineering, Tokyo, Japan) at 1 µL/cm. Membranes were blocked with 3% skim milk for 10 min, rinsed with PBS, and dried at 37°C for ≥1 h.

Recombinant streptococcal protein G (rSPG) was conjugated to 40 nm gold nanoparticles according to a modified protocol (19). After conjugation, a characteristic red shift from 522 to 530 nm confirmed successful binding (Fig. S1b).

Pretreated glass fiber conjugate pads were dispensed with gold-conjugated rSPG (10 µL/cm) and dried at 37°C for ≥1 h. Sample pads, conjugate pads, nitrocellulose membranes, and absorbent pads were laminated onto backing cards and cut into 5-mm strips.

### LFA procedure

Sensitivity was evaluated using Groups B and C, representing symptomatic and asymptomatic cases, respectively; specificity was determined using Group B. Additionally, Group D sera were used to assess cross-reactivity. Serum samples were diluted 1:20 in running buffer (PBS with 0.5% Tween 20). For each test, 80 µL of diluted serum was applied to the sample pad. After 10 min, 40 µL of running buffer was added. Results were read visually after an additional 10 min. A test was considered positive when both test and control lines were visible. Each sample was tested in triplicate. A serum sample was classified as positive if ≥2 of the 3 repetitions were positive.

### Evaluation of LFA stability

To assess stability, strips were stored with desiccant at 4°C, room temperature, or 37°C. At 1, 2, 3, 4, 8, 12, and 16 weeks after assembly, strips were tested with five Group B positive sera. Test line intensity was scored on a 10-point scale by three independent observers using the Lateral Flow Score Card. Scores ≥ 3 were considered positive.

### Statistical analysis

Sensitivity and specificity were calculated using the following formulas. Sensitivity = ∑ true glanders-positive tested individuals/∑ total glanders-positive individuals; specificity = ∑ true glanders-negative tested individuals/∑ total glanders-negative individuals. The 95% confidence intervals (CIs) for sensitivity and specificity were determined using the Jeffreys interval (20). Reproducibility was assessed based on the agreement rate across triplicates. All statistical analyses were conducted in R version 4.5.0.

## RESULTS

### Sensitivity, specificity, and reproducibility of Hcp1-, GroEL-, and WCL-LFAs

**Fig 2:**
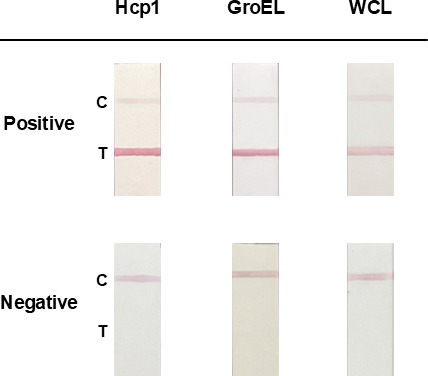
Representative results of the developed LFAs. C, control line; T, test line.

Results obtained using Group B sera are summarized in Table 1 and Table S1. Representative images of the LFA strips are shown in Fig. 2. All three LFAs demonstrated 100% diagnostic sensitivity (95% CI, 92.3%–100%). However, specificity differed across the assays. Hcp1-LFA achieved 100% specificity (95% CI, 95.2%–100%), WCL-LFA showed 98.0% specificity (95% CI, 91.2%–99.8%), and GroEL-LFA exhibited lower specificity at 90.2% (95% CI, 79.8%–96.2%). Reproducibility across triplicate testing was similarly high, with agreement rates of 98.8% for Hcp1-LFA, 98.8% for WCL-LFA, and 90.2% for GroEL-LFA.

**TABLE 1:** Sensitivity, specificity, and reproducibility of the LFAs developed with Group B sera in this study^*a*^

Antigen	% Sensitivity (CI)	% Specificity (CI)	% Agreement (No. of consistent results)
Hcp1	100 (92.3–100)	100 (95.2–100)	98.8 (81)
GroEL	100 (92.3–100)	90.2 (79.8–96.2)	90.2 (74)
WCL	100 (92.3–100)	98.0 (91.2–99.8)	98.8 (81)

^*a*^ CI, 95% confidence intervals.

Following their demonstrated high diagnostic accuracy in Group B, the results of the Hcp1- and WCL-LFAs using Group C serum samples are summarized in Table 2. The Hcp1-LFA demonstrated 100% sensitivity (95% CI, 92.3%–100%), while the WCL-LFA showed 96.8% sensitivity (95% CI, 85.9%–99.6%).

**TABLE 2:** Sensitivity of the LFAs using Group C sera^*a*^

Antigen	% Sensitivity (CI)
Hcp1	100 (92.3–100)
WCL	96.8 (85.9–99.6)

^*a*^ CI, 95% confidence intervals.

### Cross-reactivity of developed LFAs

No false-positive reactions were observed for the Hcp1-, GroEL-, or WCL-based LFAs when tested against Group D sera, indicating that none of the assays showed detectable cross-reactivity with samples collected from horses experiencing other infectious or inflammatory conditions.

### Stability of developed LFAs

Stability testing results are presented in Table S2. With the exception of a single GroEL-LFA strip stored at 37°C for 2 weeks, all test strips remained positive at every time point, and storage temperature was evaluated. For this one exception, a single observer rated the test line as negative (intensity score, 1/10), whereas the remaining observers considered the same strip positive with scores ranging from 4 to 5 out of 10. Overall, these findings demonstrate that all three LFAs retained stable performance for at least 16 weeks under a wide range of temperature conditions.

## DISCUSSION

In this study, the newly developed LFAs demonstrated excellent diagnostic performance. The Hcp1-, GroEL-, and WCL-LFAs achieved 100% sensitivity in the evaluation of symptomatic horses, while the Hcp1- and WCL-LFAs also exhibited high specificity (100% and 98.0%, respectively). Notably, the Hcp1- and WCL-LFAs maintained robust performance under conditions simulating latent or subclinical cases, with sensitivities of 100% and 96.8%, respectively. All assays remained stable for over 16 weeks at 37°C, highlighting their potential for use in field conditions.

All three LFAs exhibited 100% sensitivity using Group B serum samples. However, recent reports indicate that some *B. mallei* strains lack the *hcp1* gene due to frequent gene deletions (21), which poses a significant challenge to diagnostic methods relying on a single antigen and could result in false negatives. The inclusion of multiple antigens, such as Hcp1 and WCL, in a multiplex format may mitigate this risk and enhance overall assay reliability.

The Hcp1- and WCL-LFAs demonstrated high diagnostic sensitivity even when testing asymptomatic serum samples (Group C). Given that glanders often follows a chronic and asymptomatic course (1), the surveillance of such subclinical cases is essential for effective infection control. These results suggest that the developed LFAs are promising tools for field-based screening in endemic regions. Future studies will focus on evaluating the practical performance of these LFAs through direct field-testing in endemic areas to further validate their utility in real-world diagnostic settings.

Among the evaluated antigens, Hcp1-LFA demonstrated the highest specificity, consistent with prior studies (15, 16, 22). In contrast, GroEL-LFA and WCL-LFA showed lower specificity (90.2% and 98.0%, respectively) than that previously reported in indirect ELISA formats (GroEL, 98.0%; WCL, 100%). This discrepancy likely reflects differences in detection systems and matrix effects. LFA results are visually interpreted and qualitative, making them susceptible to subjective variation, especially for weak signals. Our stability testing exemplifies this issue, as one observer rated a GroEL-LFA strip as negative, while others scored it as positive. In addition, LFAs involve fewer washing steps and lower sample dilution compared to ELISA, increasing the likelihood of nonspecific signals. These factors, along with the limited sample size in this study, underscore the need for further validation with larger and more diverse sample sets.

GroEL-LFA also showed lower reproducibility (90.2%) compared to CFT and ID Screen ELISA. In our previous ELISA study, GroEL exhibited a higher cutoff value than Hcp1 and WCL (16), indicating borderline reactivity. Consequently, minor variations in the amount, purity, or concentration of coated GroEL during strip fabrication may have led to inconsistent results across triplicates. This variability likely reflects the small-scale, manual nature of the LFA production process.

A key feature of our LFA design is the use of rSPG as the gold-conjugated protein. Recombinant SPG is a cost-effective alternative to secondary antibodies, as it can be efficiently produced in *E. coli* and purified using a simple protocol. Moreover, SPG binds IgG from a wide range of species, including horses, donkeys, mules, and humans. Further evaluation using sera from other glanders-susceptible animals is warranted to confirm its broader applicability.

Overall, these LFAs demonstrate key advantages, including high sensitivity, high specificity, long-term stability, ease of use, and affordability. Combined, these attributes make the assays promising point-of-care diagnostic tools, particularly suitable for application in resource-limited regions where glanders remains endemic.

## Data Availability

The data that support the findings of this study are available from the corresponding author upon reasonable request.

## SUPPLEMENTAL MATERIAL

Supplemental material spectrum.00154-26-s0001.pdf Fig. S1; Tables S1 and S2.

ASM does not own the copyrights to Supplemental Material that may be linked to, or accessed through, an article. The authors have granted ASM a non-exclusive, world-wide license to publish the Supplemental Material files. Please contact the corresponding author directly for reuse.

## References

[1] World Organisation for Animal Health. 2018. Glanders and melioidosis, p 1350–1362. *In* Manual of diagnostic tests and vaccines for terrestrial animals, 8th ed. WOAH, Paris, France.

[2] Dvorak GD, Spickler AR. 2008. Glanders. J Am Vet Med Assoc 233:570–577. doi:10.2460/javma.233.4.570 18710311

[3] Derbyshire JB. 2002. The eradication of glanders in Canada. Can Vet J 43:722–726. 12240535 PMC339565

[4] Neubauer H, Sprague LD, Zacharia R, Tomaso H, Al Dahouk S, Wernery R, Wernery U, Scholz HC. 2005. Serodiagnosis of *Burkholderia mallei* infections in horses: state-of-the-art and perspectives. J Vet Med B Infect Dis Vet Public Health 52:201–205. doi:10.1111/j.1439-0450.2005.00855.x 16115091

[5] Erdemsurakh O, Ochirbat K, Gombosuren U, Tserendorj B, Purevdorj B, Vanaabaatar B, Aoshima K, Kobayashi A, Kimura T. 2020. Seroprevalence of equine glanders in horses in the central and eastern parts of Mongolia. J Vet Med Sci 82:1247–1252. doi:10.1292/jvms.20-0219 32641602 PMC7538334

[6] Singha H, Shanmugasundaram K, Tripathi BN, Saini S, Khurana SK, Kanani A, Shah N, Mital A, Kanwar P, Bhatt L, et al.. 2020. Serological surveillance and clinical investigation of glanders among indigenous equines in India from 2015 to 2018. Transbound Emerg Dis 67:1336–1348. doi:10.1111/tbed.13475 31916415

[7] Malik P, Singha H, Goyal SK, Khurana SK, Tripathi BN, Dutt A, Singh D, Sharma N, Jain S. 2015. Incidence of *Burkholderia mallei* infection among indigenous equines in India. Vet Rec Open 2:e000129. doi:10.1136/vetreco-2015-000129 26457190 PMC4594314

[8] Malik P, Khurana SK, Dwivedi SK. 2010. Re-emergence of glanders in India - report of Maharashtra state. Indian J Microbiol 50:345–348. doi:10.1007/s12088-010-0027-8 23100851 PMC3450061

[9] Tizfahm Tikmehdash H, Dehnad A, Mosavari N, Naghili Hokmabadi B, Mahmazi S. 2024. Isolation, serological and molecular methods in screening of *Burkholderia mallei* in East Azerbaijan province, Iran. Ve Res Forum 15:231–236. doi:10.30466/vrf.2024.2010651.4002 PMC11251541 39022578

[10] Nassar AFC, Chiebao DP, Fava CD, Miyashiro S, Castro V, Ogata RA, Yamamora JM, Monteiro CAS, Monteiro EJB. 2025. Histopathological and diagnostic aspects of glanders based on a case series from Brazil. J Equine Vet Sci 145:105248. doi:10.1016/j.jevs.2024.105248 39657867

[11] World Organisation for Animal Health. 2024. Infection with *Burkholderia mallei* (glanders), p 743–746. *In* Terrestrial Animal Health Code, 32nd ed. WOAH, Paris, France.

[12] Hobbs EC, Colling A, Gurung RB, Allen J. 2021. The potential of diagnostic point-of-care tests (POCTs) for infectious and zoonotic animal diseases in developing countries: technical, regulatory and sociocultural considerations. Transbound Emerg Dis 68:1835–1849. doi:10.1111/tbed.13880 33058533 PMC8359337

[13] Fritz DL, Vogel P, Brown DR, Deshazer D, Waag DM. 2000. Mouse model of sublethal and lethal intraperitoneal glanders (*Burkholderia mallei*). Vet Pathol 37:626–636. doi:10.1354/vp.37-6-626 11105952

[14] Lopez J, Copps J, Wilhelmsen C, Moore R, Kubay J, St-Jacques M, Halayko S, Kranendonk C, Toback S, DeShazer D, et al.. 2003. Characterization of experimental equine glanders. Microbes Infect 5:1125–1131. doi:10.1016/j.micinf.2003.07.004 14554254

[15] Pooja, Thapa N, Rani R, Shanmugasundaram K, Jhandai P, Rakshita, Bhattacharya TK, Singha H. 2025. Antibody isotyping and cytokine profiling in natural cases of Burkholderia mallei infection (glanders) in equines. Cytokine 185:156799. doi:10.1016/j.cyto.2024.156799 39549470

[16] Ichikawa Y, Iinuma Y, Okagawa T, Shimbo R, Enkhtuul B, Khurtsbaatar O, Kinoshita Y, Niwa H, Aoshima K, Kobayashi A, et al.. 2025. Comparison of immunogenicity of 17 *Burkholderia mallei* antigens and whole cell lysate using indirect ELISA. J Vet Med Sci 87:394–401. doi:10.1292/jvms.25-0039 40044168 PMC11964862

[17] Lewis MJ, Wagner B, Woof JM. 2008. The different effector function capabilities of the seven equine IgG subclasses have implications for vaccine strategies. Mol Immunol 45:818–827. doi:10.1016/j.molimm.2007.06.158 17669496 PMC2075531

[18] Guss B, Eliasson M, Olsson A, Uhlén M, Frej AK, Jörnvall H, Flock JI, Lindberg M. 1986. Structure of the IgG-binding regions of streptococcal protein G. EMBO J 5:1567–1575. doi:10.1002/j.1460-2075.1986.tb04398.x 3017704 PMC1166981

[19] Xu R, Feng J, Hong Y, Lv C, Zhao D, Lin J, Lu K, Li H, Liu J, Cao X, et al.. 2017. A novel colloidal gold immunochromatography assay strip for the diagnosis of schistosomiasis japonica in domestic animals. Infect Dis Poverty 6:84. doi:10.1186/s40249-017-0297-z 28388965 PMC5384140

[20] Signorell A. 2025. DescTools: tools for descriptive statistics. 10.32614/CRAN.package.DescTools.

[21] Charron P, Gao R, Chmara J, Hoover E, Nadin-Davis S, Chauvin D, Hazelwood J, Makondo K, Duceppe MO, Kang M. 2023. Influence of genomic variations on glanders serodiagnostic antigens using integrative genomic and transcriptomic approaches. Front Vet Sci 10:1217135. doi:10.3389/fvets.2023.1217135 38125681 PMC10730941

[22] Wagner GE, Berner A, Lipp M, Kohler C, Assig K, Lichtenegger S, Saqib M, Müller E, Trinh TT, Gad AM, et al.. 2023. Protein microarray-guided development of a highly sensitive and specific dipstick assay for glanders serodiagnostics. J Clin Microbiol 61:e0123422. doi:10.1128/jcm.01234-22 36541753 PMC9879090

