# Supplementary material for: Development of lateral flow assays for the rapid diagnosis of glanders in equids
Source: Microbiol Spectr. 2026 Jul 10;14(8):e00154-26. doi: 10.1128/spectrum.00154-26 (PMC13436117; doi:10.1128/spectrum.00154-26)
Supplement: Supplementary file 1 [file spectrum.00154-26-s0001.pdf]

Supplemental table.2 The intensity score of the test line bands in stability evaluation

Observer #1	Serum						Serum						Serum				
Hcp1	#1	#2	#3	#4	#5		#1	#2	#3	#4	#5		#1	#2	#3	#4	#5
4°C_1w	10	10	10	10	10	RT_1w	9	10	10	10	10	37°C_1w	5	10	10	10	10
4°C_2w	7	7	8	8	8	RT_2w	7	8	8	8	8	37°C_2w	7	8	8	9	8
4°C_3w	7	8	8	9	7	RT_3w	6	8	7	7	7	37°C_3w	7	9	6	8	8
4°C_4w	6	7	7	7	7	RT_4w	7	8	8	8	8	37°C_4w	8	8	8	9	9
4°C_8w	7	7	7	7	7	RT_8w	7	7	7	7	7	37°C_8w	7	8	8	8	8
4°C_12w	7	8	7	8	6	RT_12w	7	7	7	7	7	37°C_12w	6	6	5	6	6
4°C_16w	7	7	7	7	7	RT_16w	8	7	7	5	6	37°C_16w	7	5	6	7	6

	Serum						Serum						Serum				
GroEL	#1	#2	#3	#4	#5		#1	#2	#3	#4	#5		#1	#2	#3	#4	#5
4°C_1w	7	7	7	7	7	RT_1w	7	7	6	7	8	37°C_1w	8	8	7	8	8
4°C_2w	6	6	5	5	6	RT_2w	6	7	5	6	6	37°C_2w	6	7	1	5	6
4°C_3w	7	7	6	6	7	RT_3w	6	7	5	6	6	37°C_3w	7	8	6	7	7
4°C_4w	5	6	6	6	5	RT_4w	6	7	6	7	6	37°C_4w	6	7	6	7	7
4°C_8w	6	6	5	6	6	RT_8w	6	6	6	7	6	37°C_8w	5	7	5	6	6
4°C_12w	6	6	5	6	5	RT_12w	7	7	5	7	6	37°C_12w	6	6	5	6	5
4°C_16w	7	7	5	6	6	RT_16w	6	6	5	6	6	37°C_16w	5	5	5	6	5

	Serum						Serum						Serum				
WCL	#1	#2	#3	#4	#5		#1	#2	#3	#4	#5		#1	#2	#3	#4	#5
4°C_1w	8	7	7	7	5	RT_1w	8	6	8	8	6	37°C_1w	7	8	8	8	5
4°C_2w	6	8	7	7	5	RT_2w	6	7	7	6	5	37°C_2w	5	6	6	6	4
4°C_3w	7	8	7	7	5	RT_3w	7	9	8	7	5	37°C_3w	7	7	5	7	7
4°C_4w	6	5	6	5	8	RT_4w	5	7	4	6	5	37°C_4w	7	7	6	7	5
4°C_8w	5	6	6	6	5	RT_8w	6	6	6	6	5	37°C_8w	5	7	6	6	6
4°C_12w	7	6	7	7	5	RT_12w	7	6	7	7	6	37°C_12w	7	6	5	6	5
4°C_16w	7	6	6	7	5	RT_16w	7	5	6	6	5	37°C_16w	5	5	6	6	5

Observer #2	Serum						Serum						Serum				
Hcp1	#1	#2	#3	#4	#5		#1	#2	#3	#4	#5		#1	#2	#3	#4	#5
4°C_1w	9	9	9	9	9	RT_1w	10	10	10	10	10	37°C_1w	9	10	10	10	10
4°C_2w	8	8	8	8	8	RT_2w	9	10	9	9	9	37°C_2w	8	9	9	9	8
4°C_3w	8	8	8	9	8	RT_3w	7	9	8	8	8	37°C_3w	8	9	8	9	8
4°C_4w	7	8	8	8	7	RT_4w	8	9	9	9	9	37°C_4w	8	8	8	9	9
4°C_8w	7	8	8	8	7	RT_8w	8	8	8	8	8	37°C_8w	8	9	9	9	8
4°C_12w	7	7	7	7	6	RT_12w	8	8	8	8	8	37°C_12w	8	8	7	8	8
4°C_16w	8	8	8	8	8	RT_16w	8	8	8	7	8	37°C_16w	7	7	6	8	7

	Serum						Serum						Serum				
GroEL	#1	#2	#3	#4	#5		#1	#2	#3	#4	#5		#1	#2	#3	#4	#5
4°C_1w	9	9	8	8	9	RT_1w	8	9	8	9	9	37°C_1w	8	8	7	8	8
4°C_2w	7	7	7	6	7	RT_2w	7	7	6	7	7	37°C_2w	7	7	5	7	6
4°C_3w	7	7	7	6	7	RT_3w	7	8	6	7	7	37°C_3w	6	8	6	7	7
4°C_4w	7	8	7	7	7	RT_4w	6	8	6	8	6	37°C_4w	6	7	6	7	7
4°C_8w	8	8	7	8	8	RT_8w	8	8	7	8	8	37°C_8w	6	8	6	6	6
4°C_12w	8	8	7	8	8	RT_12w	7	7	5	8	7	37°C_12w	6	6	5	6	6
4°C_16w	8	8	7	8	8	RT_16w	7	7	5	7	7	37°C_16w	7	8	7	7	7

	Serum						Serum						Serum				
WCL	#1	#2	#3	#4	#5		#1	#2	#3	#4	#5		#1	#2	#3	#4	#5
4°C_1w	9	9	9	9	8	RT_1w	9	9	9	9	7	37°C_1w	8	9	9	9	6
4°C_2w	9	10	9	9	8	RT_2w	7	9	9	7	6	37°C_2w	7	9	8	8	7
4°C_3w	9	10	9	9	6	RT_3w	9	10	9	9	6	37°C_3w	9	9	7	9	9
4°C_4w	8	6	6	6	9	RT_4w	7	8	6	8	6	37°C_4w	8	9	9	9	7
4°C_8w	6	8	7	7	6	RT_8w	8	8	8	7	7	37°C_8w	6	8	8	8	7
4°C_12w	8	7	7	7	6	RT_12w	9	7	9	9	7	37°C_12w	8	7	7	8	7
4°C_16w	8	7	7	8	6	RT_16w	8	9	7	8	7	37°C_16w	7	7	8	8	7

Observer #3	Serum						Serum						Serum				
Hcp1	#1	#2	#3	#4	#5		#1	#2	#3	#4	#5		#1	#2	#3	#4	#5
4°C_1w	10	10	10	10	10	RT_1w	10	10	10	10	10	37°C_1w	10	10	10	10	10
4°C_2w	7	8	8	8	8	RT_2w	7	8	7	7	7	37°C_2w	7	8	8	8	7
4°C_3w	8	9	8	9	7	RT_3w	6	7	6	6	6	37°C_3w	7	8	7	8	8
4°C_4w	7	9	9	8	8	RT_4w	6	7	7	7	7	37°C_4w	8	8	8	8	8
4°C_8w	9	9	9	9	9	RT_8w	7	7	7	7	7	37°C_8w	8	8	8	8	8
4°C_12w	8	9	8	8	7	RT_12w	7	7	7	7	7	37°C_12w	7	7	6	7	7
4°C_16w	8	8	8	8	8	RT_16w	7	7	7	6	7	37°C_16w	7	6	7	7	7
	Serum						Serum						Serum				
GroEL	#1	#2	#3	#4	#5		#1	#2	#3	#4	#5		#1	#2	#3	#4	#5
4°C_1w	8	8	8	7	8	RT_1w	7	8	7	7	7	37°C_1w	8	8	7	8	8
4°C_2w	7	7	6	5	7	RT_2w	6	6	5	6	6	37°C_2w	7	7	4	5	5
4°C_3w	8	8	7	7	8	RT_3w	6	7	6	7	7	37°C_3w	6	7	5	6	7
4°C_4w	6	8	7	7	6	RT_4w	6	7	6	6	6	37°C_4w	6	7	5	7	7
4°C_8w	8	8	6	8	8	RT_8w	7	7	7	7	7	37°C_8w	6	8	6	6	6
4°C_12w	8	8	6	7	7	RT_12w	7	7	4	7	6	37°C_12w	6	6	5	7	6
4°C_16w	8	8	5	7	7	RT_16w	7	7	5	7	7	37°C_16w	7	7	6	7	6
	Serum						Serum						Serum				
WCL	#1	#2	#3	#4	#5		#1	#2	#3	#4	#5		#1	#2	#3	#4	#5
4°C_1w	8	8	8	8	7	RT_1w	8	8	8	8	8	37°C_1w	8	8	8	8	5
4°C_2w	8	9	8	8	6	RT_2w	6	8	8	8	5	37°C_2w	7	8	7	7	5
4°C_3w	8	9	9	8	5	RT_3w	8	8	8	8	4	37°C_3w	8	8	6	8	8
4°C_4w	6	5	6	5	8	RT_4w	6	7	5	7	5	37°C_4w	8	8	8	8	6
4°C_8w	5	8	7	8	6	RT_8w	7	7	7	6	6	37°C_8w	6	8	7	7	7
4°C_12w	8	7	8	8	6	RT_12w	8	7	8	8	6	37°C_12w	7	6	6	7	6
4°C_16w	8	7	8	8	6	RT_16w	8	7	8	8	7	37°C_16w	7	6	7	7	6

A single false-negative result is highlighted. RT: room temperature.
